# Verification of CT/MRI imaging protocol compliance for radiotherapy

**DOI:** 10.1002/acm2.70246

**Published:** 2025-09-09

**Authors:** Yunfei Hu, James D. Rijken, Marius Arnesen

**Affiliations:** ^1^ Icon Cancer Centre Gosford Gosford New South Wales Australia; ^2^ Icon Cancer Centre Windsor Gardens Windsor Gardens South Australia Australia; ^3^ Icon Cancer Centre Toowoomba Toowoomba Queensland Australia

**Keywords:** CT, MRI, radiotherapy QA, Scripting

## Abstract

**Introduction:**

The role of imaging in radiotherapy is becoming increasingly important. Verification of imaging parameters prior to treatment planning is essential for safe and effective clinical practice.

**Methods:**

This study described the development and clinical implementation of ImageCompliance, an automated, GUI‐based script designed to verify and enforce correct CT and MRI parameters during radiotherapy planning.

**Results:**

Since its deployment, ImageCompliance has processed more than 48,000 CT and MRI studies. The integration of the centralized database eliminated the need for manual uploads, reducing workflow inefficiencies. The multi‐tier warning system facilitated timely identification of deviations from protocol, thereby supporting clinical decision‐making and ensuring protocol adherence.

**Conclusion:**

The clinical implementation of ImageCompliance safeguards geometric and dosimetric accuracy, informs margin selection, and enhances governance over imaging protocols. It represents an effective strategy for the verification of CT/MRI imaging protocol compliance for radiotherapy.

## 1 INTRODUCTION

Radiotherapy requires an accurate understanding of both the spatial configuration and dosimetric properties of the target. X‐ray computed tomography (CT) scanners are widely used in radiotherapy planning because they not only delineate internal and external anatomical contours but also create a density map (in the form of electron or mass densities) that is crucial for precise x‐ray beam penetration calculations.^1^, ^2^ More recently, magnetic resonance imaging (MRI) has emerged as a vital imaging modality in radiotherapy, owing to its superior soft tissue contrast and non‐ionizing characteristics.^3^, ^4^

Despite well‐established quality assurance (QA) standards for CT and MRI scanners^5^, ^6^ and the development of dedicated CT and MRI protocols for radiotherapy patients,^7^, ^8^ verifying that the correct CT or MRI protocol has been employed for clinical planning is often overlooked, as indicated in the literature.^7^, ^9^ Meanwhile, it has been pointed out that clinically, several imaging parameters can be changed together, potentially creating a cumulative effect on both image quality and HU consistency.^10^ Although some studies have explored the application of failure mode analysis to the entire treatment chain,^11^, ^12^ including imaging, existing research of errors in radiotherapy has predominantly concentrated on the delivery side, undermining the potential role CT and MRI imaging in cumulative uncertainty throughout the treatment chain.^13^, ^14^, ^15^

At the authors’ institution, composed of multiple regional centers, many facilities do not have dedicated CT or MRI scanners. Instead, they rely on collaborations with local radiology departments, where radiotherapy patients are scanned using protocols tailored for radiotherapy. Under these circumstances, the risk of using imaging parameters that have not been commissioned and endorsed for clinical use is elevated. The use of incorrect imaging parameters, or more seriously, incorrect imaging protocols, can lead to severe consequences, such as geometric misses and inaccurate dose calculations. For example, studies have indicated that for a given CT scanner, variations in tube voltage can exhibit significant effects on measured CT numbers,^7^, ^10^, ^16^, ^17^, ^18^, ^19^ which, in turn, impacts dose calculation accuracies. Like previous work by the authors in performing QA checks on breathing traces before 4DCT reconstruction,^20^ it is imperative to implement a QA measure that verifies the parameters used during image acquisition prior to treatment planning. While research has highlighted the necessity of such measures,^9^ reports on their clinical implementation are scarce.

To address this challenge, the authors have developed a script‐based individual CT/MRI QA tool for radiotherapy patients, called “ImageCompliance.” The purpose of this study is to provide an overview of ImageCompliance, describe its clinical implementation, and review selected clinical near‐miss incidents identified by the script.

## 2 MATERIALS AND METHODS

The graphic user interface (GUI)‐based ImageCompliance script was written in C# to be run through the Eclipse treatment planning system (Varian Medical Systems, Palo Alto, USA). The script was integrated within the Aria Record & Verify system (Varian Medical Systems, Palo Alto, USA). ImageCompliance went through two major versions. Regardless of the version, the core function of ImageCompliance is to import a DICOM slice from a clinical CT or MR dataset, extract the relevant DICOM tags to memory, and analyze it against predefined criteria.

The first version, ImageCompliance V1.0, required a reference scan for each commissioned protocol. During the check process, an RT first loaded the reference file, followed by loading a random slice from the patient scan. The script then automatically compared a series of DICOM tags between the patient and the reference slices against predefined criteria. ImageCompliance V1.0 utilized a single‐tier warning system. Parameters with values outside the predefined tolerance would be labelled as “Fail,” immediately terminating the planning process from proceeding until the warning was acknowledged or resolved. Notably, in ImageCompliance V1.0, due to the lack of reference data, the tolerance range for tube current and CTDIvol was set arbitrarily (± 100%) during the initial deployment.

Over a 30‐month period, ImageCompliance V1.0 ingested approximately 18 000 CT datasets and 3000 MRI datasets. At the conclusion of the clinical deployment, the ImageCompliance script went through a major functional upgrade, referred to as “ImageCompliance V2.0.” The parameters checked by ImageCompliance V2.0, along with their DICOM tag, reference value, tolerance, and warning tier, are listed in Table 1.

**TABLE 1:** Details of parameters checked or reported by ImageCompliance V2.0.

Data	DICOM Tag	Reported for	Reference value	Tolerance	Warning tier
Patient ID	0010,0020	CT & MR	NA	NA	Report only
Patient name	0010,0010	CT & MR	NA	NA	Report only
Study date	0008,0020	CT & MR	NA	NA	Report only
Modality	0008,0060	CT & MR	NA	NA	Report only
Body region	0018,0015 & 0008,1030	CT & MR	NA	NA	Report only
Slice thickness	0018,0050	CT & MR	1.0 mm for stereotactic scans; Protocol‐specific for non‐stereotactic scans, typically 2.0–2.5 mm	≤	Fail
kVp	0018,0060	CT	Protocol‐specific, typically 120	=	Fail
Data collection diameter	0018,0090	CT	Protocol‐specific, typically 500 mm	≤	Fail
Reconstruction diameter	0018,1100	CT	Protocol‐specific, typically 700 mm	≤	Fail
Gantry tilt	0018,1120	CT	0	=	Fail
Tube current	0018,1151	CT	Scanner model‐specific, determined from historical data	Within 95% confidence interval	Physics review^a^
Filter type	0018,1160	CT	Protocol‐specific	=	Fail
Focal spot	0018,1190	CT	Protocol‐specific	=	Fail
Convolution Kernel	0018,1210	CT	Protocol‐specific	=	Fail
Patient position	0018,5100	CT	Protocol‐specific, typically Headfirst Supine	=	Warning
CTDIvol	0018,9345	CT	Scanner model‐specific, determined from historical data	Within 95% Confidence Interval	Physics review
Exposure time	0018,1150	CT	NA	NA	Report only
Protocol name	0018,1030	CT	NA	NA	Report only
2D distortion correction	0008,0008	MR	Yes	=	Fail
Manufacturer	0008,0070	MR	Protocol‐specific	=	Fail
Model	0008,1090	MR	Protocol‐specific	=	Fail
Serial number	0018,1000	MR	Protocol‐specific	=	Fail
Series description	0008,103E	MR	Protocol‐specific	=	Fail^b^
3D distortion Correction	0008,0008 & 0008,103E	MR	Yes	=	Fail^b^ or Physics Review^c^
Sequence weighting	0008,103E	MR	Protocol‐specific	=	Fail^b^
Scanning sequence	0018,0020	MR	Protocol‐specific	=	Fail^b^
Voxel dimension	0028,0030	MR	Protocol‐specific	≤	Fail^b^
FOV size	0028,0030, 0028,0011 & 0028,0010	MR	NA	NA	Report only
Phase encode direction	0018,9034	MR	NA	NA	Report only
Pixel bandwidth	0018,0095	MR	NA	NA	Report only

^a^ Check of tube current is only applicable to GE scanners, as they do not report CTDIvol.

^b^ For cranial stereotactic treatment only.

^c^ For non‐cranial stereotactic treatment only.

ImageCompliance V2.0 automatically compares a series of DICOM tags in the clinical patient scan to a set of centrally stored reference values, against predefined criteria. Additionally, discrepancies are flagged in a multi‐tier warning system as a “Warning,” “Physics Review,” or “Fail” depending on their potential implications. Parameters with direct implications for dosimetric or geometric accuracy, such as tube voltage and slice thickness for CT images,^7^, ^10^ and the application of 2D or 3D distortion correction for MRIs,^9^ are designated “Fail” when they fall out of tolerance, halting the planning process until the warning is acknowledged or resolved. Alternatively, metrics related to the scan dose, such as CTDIvol and tube current, only trigger “Physics Review” if they exceed the tolerance. Because these parameters are deemed to have minimal impact on treatment planning accuracy, the planning workflow may continue while a justification is obtained. Lastly, deviations from the standard Headfirst Supine patient orientation trigger a “Warning,” prompting radiation therapists to verify acquisition posture without interrupting the planning process, whereas physicists do not need to be involved in the process. Moreover, certain criteria are treatment‐technique dependent, with more stringent alert levels applied to MRI scans intended for cranial stereotactic planning due to the requirement for tighter margins.

In ImageCompliance V2.0, scanner model‐specific tolerance values for CTDIvol and tube current were established in replacement of the prior arbitrary tolerances. These values, listed in Table 2, were derived from the 95% confidence interval of historical data collected by ImageCompliance V1.0.

**TABLE 2:** Scanner model‐specific tolerances for CTDIvol and tube current, derived from historical data.

Scanner type	Protocol	95% confidence interval
CTDIvol (mGy)		
Siemens—go. Series scanners (go.Sim, go.Up, etc.)	Head Non‐stereo	0–77
	Head Stereo	0–100
	Body Non‐stereo	0–24
	Body Stereo	0–26
	Extremity	0–29
Siemens—other scanners	Head Non‐stereo	0–79
	Head Stereo	0–138
	Body Non‐stereo	0–34
	Body Stereo	0–33
	Extremity	0–41
Canon & Toshiba scanners	Head Non‐stereo	0–88
	Head Stereo	0–80
	Body Non‐stereo	0–33
	Body Stereo	0–35
	Extremity	0–18
Tube current (mAs)		
GE scanners	Head Non‐stereo	0–400
	Head Stereo	0–400
	Body Non‐stereo	0–300
	Body Stereo	0–300

**FIGURE 1:**
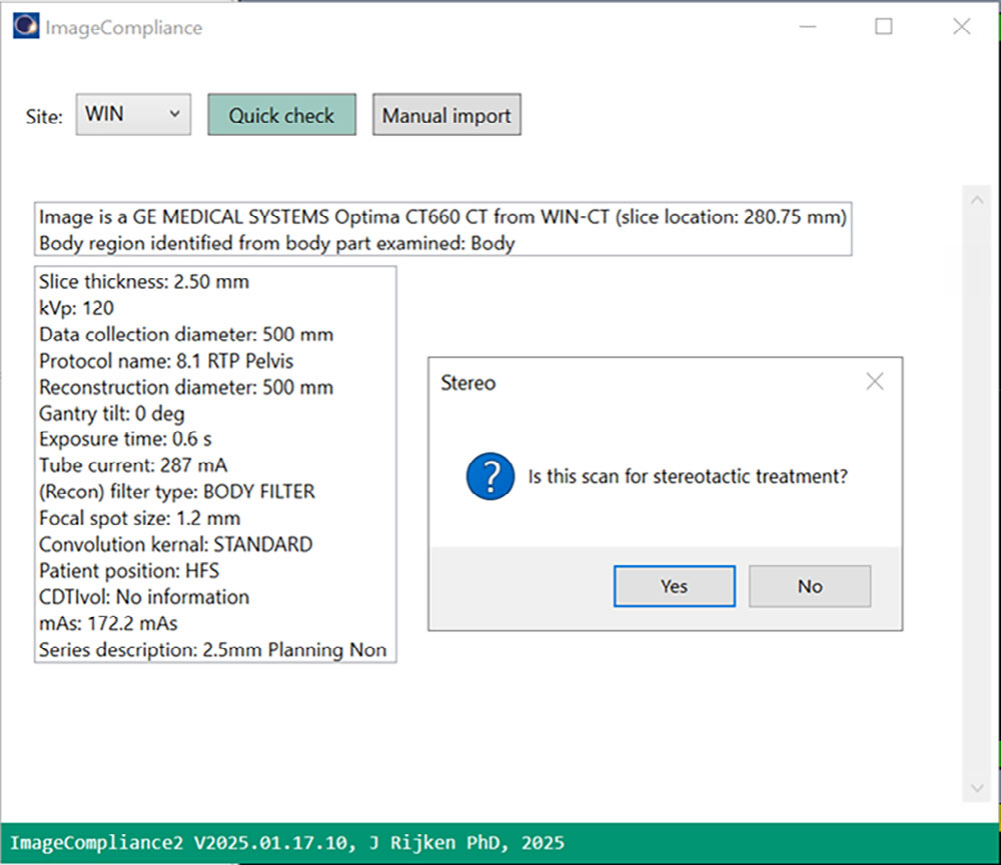
GUI of ImageCompliance V2.0.

The GUI of ImageCompliance V2.0 is shown in Figure 1.

**FIGURE 2:**
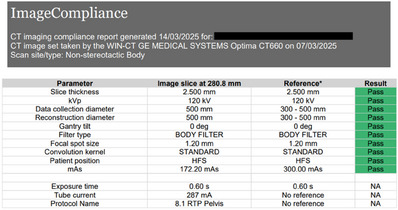
Sample CT QA report generated by ImageCompliance V2.0.

A sample CT report generated by ImageCompliance V2.0 is shown in Figure 2.

**FIGURE 3:**
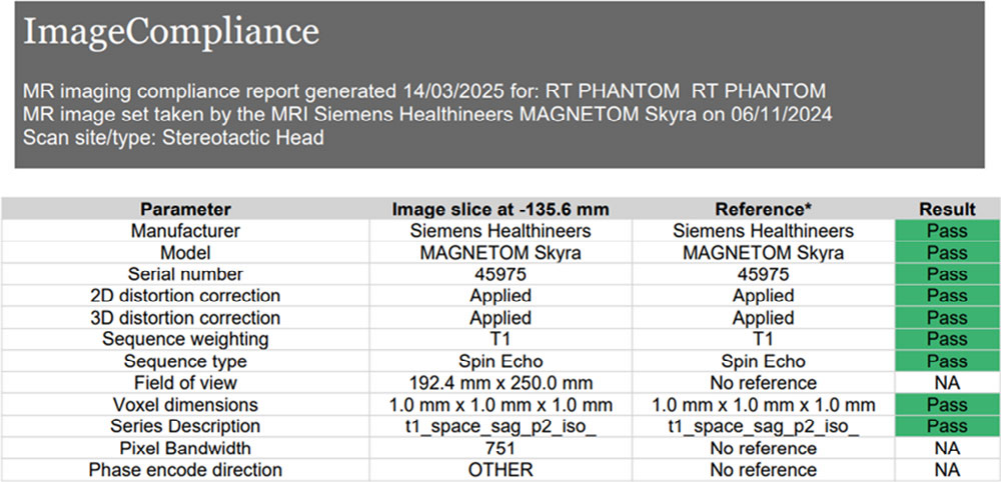
Sample MRI QA report generated by ImageCompliance V2.0.

A sample MR report generated by ImageCompliance V2.0 is provided in Figure 3.

All reference values in ImageCompliance V2.0 are maintained in the central CSV file, offering a scalable, efficient framework for storing, tracking, and updating scan parameters across the distributed network of CT and MRI sites within the authors’ institution. This centralized approach enhances governance over the QA process and facilitates the rapid identification of sites with outlier protocol settings. Additionally, as reference values for multiple parameters are based on historical data, minimal user input is required when establishing baselines. Therefore, the central CSV can be easily expanded to include new scanners or updated whenever there are changes to existing image protocols.

**FIGURE 4:**
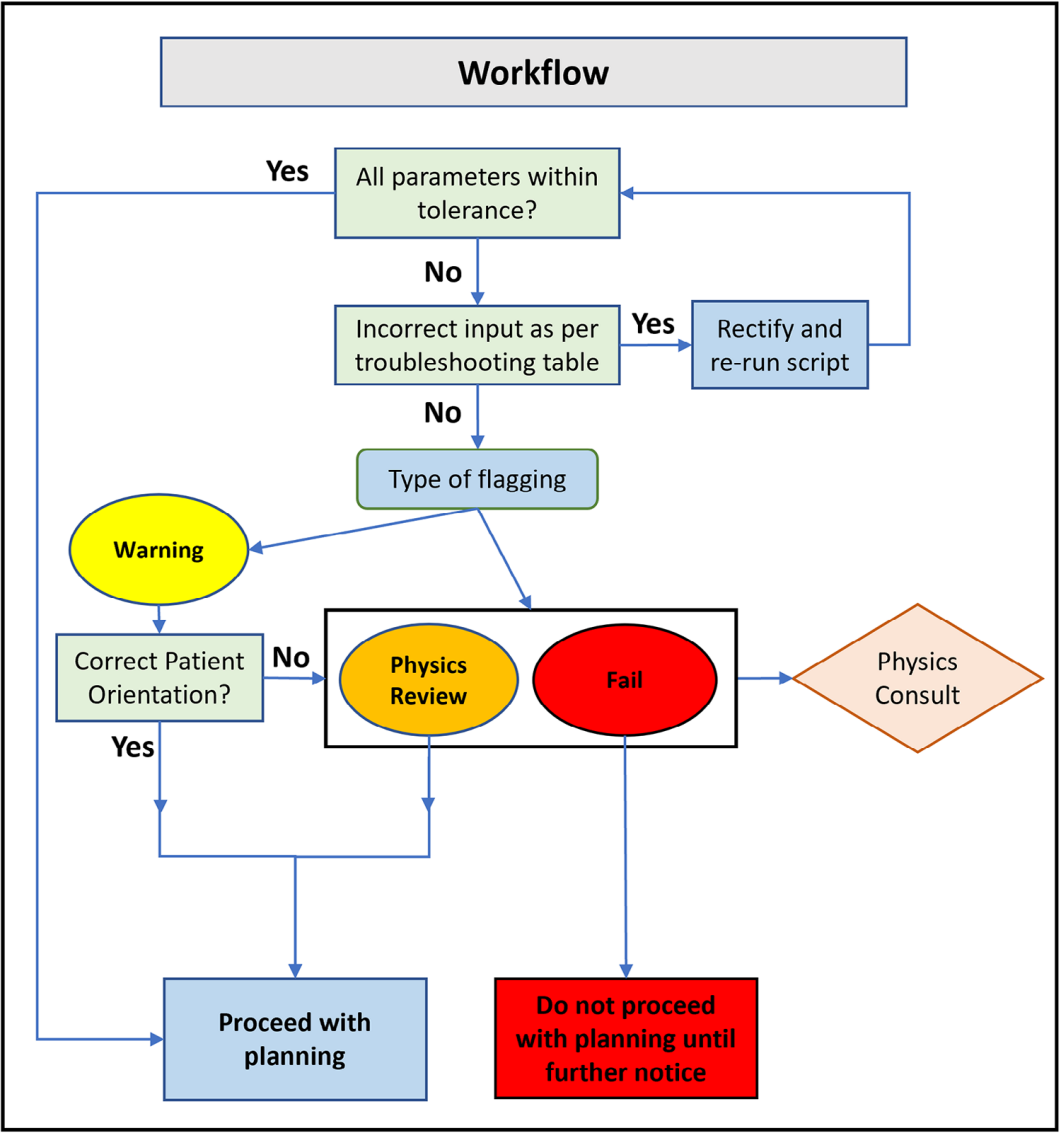
Workflow for addressing different tires of warnings generated by ImageCompliance V2.0.

The use of ImageCompliance V2.0 prior to the import of a CT/MRI dataset is enforced via the CarePath workflow in Aria. Radiation therapists must follow the prescribed corrective actions for each warning tier and are encouraged to lodge any user‐induced errors in the department's risk register, allowing subsequent rectification. A work instruction was released in conjunction with ImageCompliance V2.0 to facilitate clinical implementation. The same work instruction also entails actions that can be taken upon different tiers of warnings, such as repeating CT reconstruction with the correct slice thickness, retrospectively applying distortion correction for an MRI scan, or performing a rescan of the patient if no other measures are available. Figure 4 illustrates the workflow for responding to each warning tier generated by ImageCompliance for CT scans.

## 3 RESULTS

**FIGURE 5:**
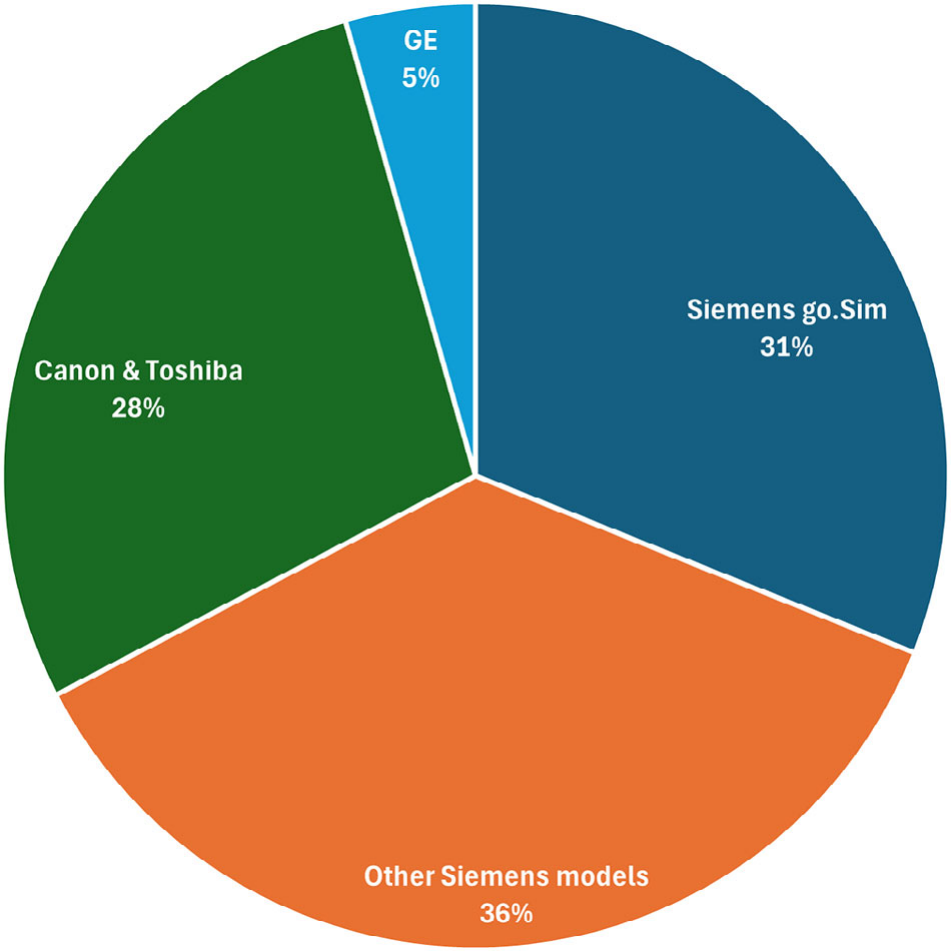
Distribution of CT datasets by scanner model.

**FIGURE 6:**
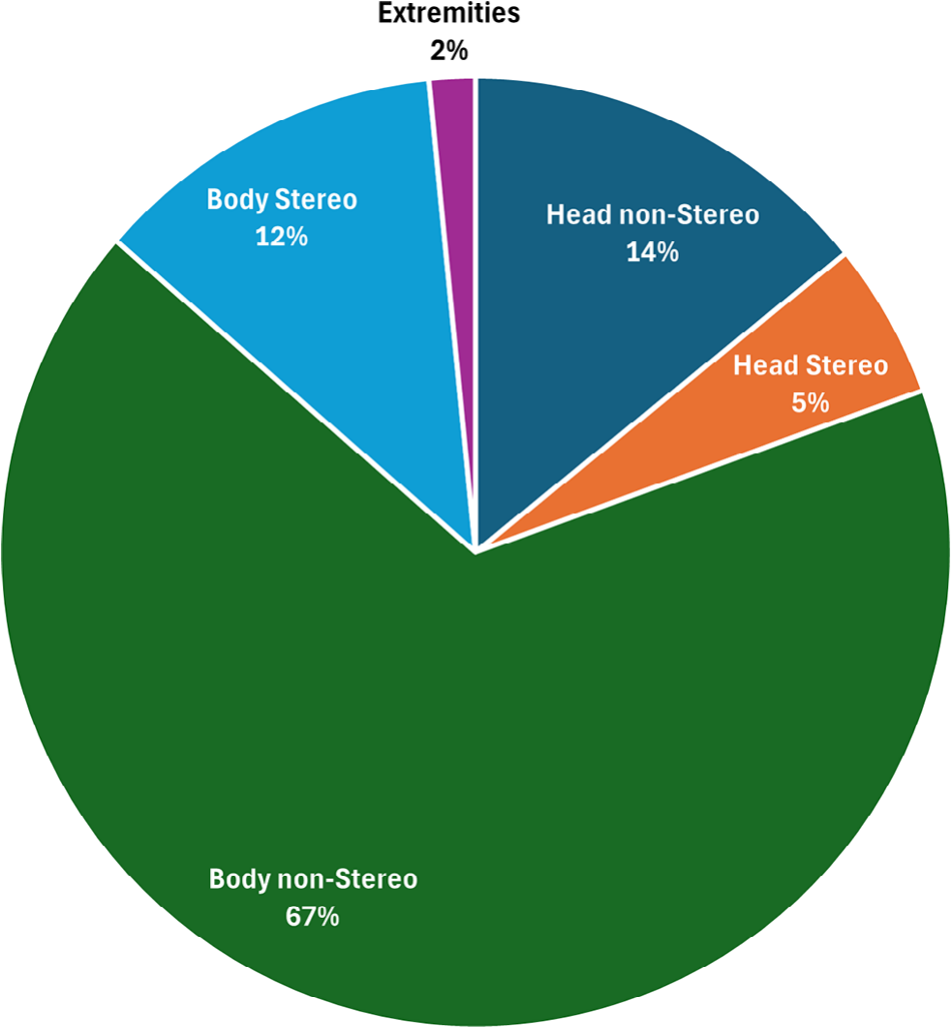
Distribution of CT datasets by anatomical area.

ImageCompliance V2.0 was first deployed on February 15, 2024. From its initial implementation up until July 28, 2025, a total of 27 897 CT datasets and 5688 MRI datasets have been checked. Figure 5 illustrates the distribution of CT datasets by scanner model, while Figure 6 presents their distribution by anatomical site.

Over a 6‐month period spanning January and June 2025, out of the 12 125 datasets examined by ImageCompliance v2.0, a total of 20 near‐miss incidents were reported by radiation therapists, representing an incident rate of 0.16%. Details of these incidents are listed in Table 3. The frequency of each type of failure in Table 3 is calculated as the number of near‐miss incidents of a particular type of failure divided by the total number of reported near‐miss incidents for CT or MRI.

**TABLE 3:** Types of the most common failures identified by ImageCompliance V2.0 and their associated frequencies.

Type of failure	Frequency	Warning tier
CT		
Incorrect tube voltage (kVp)	35%	Fail
Incorrect slice thickness	30%	Fail
Incorrect convolution kernel	25%	Fail
Other	10%	Physics review
MRI		
Incorrect distortion correction	80%	Fail
Incorrect sequence weighting	20%	Fail

## 4 DISCUSSION

Although QA systems and standards for CT and MRI scanners and simulation processes^5^, ^6^ have been well established, there remains no consensus on how to ensure that the correct imaging parameters have been utilized during simulation. This issue is particularly pertinent when a radiotherapy center does not have its own dedicated CT or MRI scanner but relies on a collaboration with external providers to acquire planning scans. Consequently, the authors have deployed ImageCompliance to verify that appropriate scanning parameters, especially those directly associated with dosimetric and geometric accuracy, have been utilized in clinical radiotherapy scans.

One of the most common issues identified by the ImageCompliance script is the alteration of tube voltage settings on a CT scan, whether intentional or inadvertent. It is well recognized that tube voltage directly affects the CT number and, consequently, the electron density (ED).^16^, ^17^, ^18^, ^19^, ^21^, ^22^ Therefore, it is essential to ensure that a clinical scan is performed with the same tube voltage that was used during commissioning to establish the CT‐ED curve. Nonetheless, in radiology departments, it is common practice for technicians to adjust the tube voltage on the spot to minimize the radiation dose to the patient. Even when RTs are present during simulation, such changes in tube voltage may sometimes go unnoticed, potentially leading to errors in dose calculation if not detected in time by the ImageCompliance script.

Another common issue for CT scans is the variability in slice thickness, a parameter that can be easily modified by the operator during scan acquisition. Notably, both manual^23^ and automatic^24^ contouring processes have identified slice thickness as a critical factor influencing contour quality and consistency. Specifically, for auto‐contouring, increasing the slice thickness has been demonstrated to have a significantly more pronounced effect on contouring quality compared to the effect on CT dose, with a greater impact observed for smaller structures.^24^ In stereotactic treatment, where targets may be very small, a retrospective cohort study revealed that increasing the slice thickness from 1 to 2  or 3 mm resulted in 3% and 13% of lesions being missed, respectively.^25^ Therefore, it is crucial to ensure that the dataset is acquired using the correct slice thickness, particularly for radiotherapy treatments that demand high precision, such as stereotactic treatments. The ImageCompliance script has proven both useful and efficient in identifying and preventing such errors with minimal human intervention.

At the time this article was written, the MRI module of the ImageCompliance script was still in its early form, validating only a limited set of acquisition parameters. Despite so, a common issue identified by the script was the inconsistent application of distortion correction. MRI distortion correction enhances the accuracy of anatomical and functional analyses by addressing geometric inaccuracies caused by magnetic field inhomogeneities and gradient non‐linearities.^26^ This correction not only improves the accuracy of structure delineation but also plays a critical role in image registration.^27^ Its application is especially important in clinical scenarios such as stereotactic and functional neurosurgery, where accuracy within 1–2 mm is required. However, the application of geometric distortion correction is sometimes not automatically linked to the protocol and must be manually enabled. The ImageCompliance script has been shown to effectively detect MRI scans where distortion correction has not been applied, thereby preventing the use of images with significant geometric uncertainties that might otherwise be overlooked during visual inspection.

In addition to its QA function, the significant amount of clinical data collected by ImageCompliance V2.0 underpins the development and optimization of CT and MRI protocols dedicated to radiotherapy purposes. A project is currently underway to standardize the CT and MRI protocols across different centers within the author's institution, aiming to eliminate inter‐departmental variations for protocols of the same anatomical area and ensure consistent image quality. Such standardization mitigates user‐related errors, facilitates the implementation of automated contouring workflows,^28^ simplifies protocol management, and paves the way for a universal CT calibration curve in the treatment planning system. The findings of this initiative will be reported in a future study once available.

## 5 CONCLUSION

This study described the development and clinical implementation of an automated QA tool, ImageCompliance. By extracting and analyzing DICOM tags prior to importing a CT or MRI dataset for radiotherapy planning, ImageCompliance ensures the correct use of key imaging parameters, such as tube voltage, slice thickness, and the application of distortion correction, prior to their use in contouring and treatment planning. This proactive verification helps mitigate the risk of image‐related errors and enhances clinical efficiency. The findings of this study offer novel insights into individual CT/MRI protocol QA for radiotherapy patients, an aspect that is often overlooked and yet can introduce significant errors into the radiotherapy treatment chain.

## AUTHOR CONTRIBUTIONS

Yunfei Hu, James D. Rijken, and Marius Arnesen conceived and designed the project. James D. Rijken acquired the data. Yunfei Hu and Marius Arnesen analyzed and interpreted the data. Yunfei Hu, James D. Rijken, and Marius Arnesen wrote and reviewed the article.

## CONFLICT OF INTEREST STATEMENT

The authors declare no conflicts of interest.

## Data Availability

Authors will share data upon reasonable request to the corresponding author.
